# Identifying sarcopenia and sarcopenic obesity in a lower extremity arthroplasty clinical setting: a pragmatic pilot study

**DOI:** 10.1016/j.tjfa.2025.100125

**Published:** 2026-01-31

**Authors:** K. Godziuk, I. Hollyer, G. Loughran, N.J. Giori

**Affiliations:** a Department of Physical Therapy and Rehabilitation Science, School of Medicine, University of California, San Francisco, CA, USA; b Department of Agricultural, Food and Nutritional Science, Faculty of Agricultural, Life and Environmental Sciences, University of Alberta, Edmonton, AB, Canada; c Department of Orthopaedic Surgery, Boston Children's Hospital, Boston, MA, USA; d Department of Orthopaedic Surgery, Vanderbilt University, Nashville, TN, USA; e Department of Orthopaedic Surgery, School of Medicine, Stanford University, Stanford, CA, USA; f VA Palo Alto Health Care System, Palo Alto, CA, USA

**Keywords:** Arthroplasty, Total joint replacement, Obesity, Sarcopenia

## Abstract

Sarcopenia and sarcopenic obesity may increase surgical complications and impact recovery and function after total joint arthroplasty (TJA). We assessed the feasibility of identifying these conditions in an orthopedic practice setting using published consensus criteria. Patients in a lower extremity TJA clinic were assessed for sarcopenia and sarcopenic obesity using EWGSOP2 and ESPEN/EASO diagnostic frameworks, respectively. Low strength testing involved maximal handgrip strength (HGS) and number of chair sit-to-stands in 30 seconds (CSTS). Same day dual-energy x-ray absorptiometry (DXA) testing was used to assess for low muscle mass (i.e. appendicular lean soft tissue) in patients with low strength. One hundred-one of a possible 128 patients were assessed in clinic (93% male, mean age 69.6±8.9 years and BMI 31.7±7.9 kg/m^2^). HGS was completed in 99% of screened patients; only 44.5% completed CSTS due to joint pain and balance limitations. Thirty-nine patients had low strength and were recommended for DXA. In 16 patients who completed DXA, 3 had sarcopenia and 5 had sarcopenic obesity. Screening for sarcopenia and sarcopenic obesity was challenging to complete in all patients during routine clinic flow with dedicated personnel. Despite our pragmatic approach and limited screening completion in all patients, we identified sarcopenic and sarcopenic obesity in 6.25% of patients. This is likely a lower bound for the true prevalence but suggests an opportunity to assess and intervene for these conditions before surgery to improve total joint arthroplasty outcomes.

## 1 Introduction

There is growing awareness of the negative implications of unidentified sarcopenia in the orthopedic surgical setting, particularly in individuals with advanced knee or hip osteoarthritis (OA) who are seeking arthroplasty[1]. Sarcopenia is defined as an accelerated loss of skeletal muscle mass and strength associated with aging and other health conditions[2,3]. Sarcopenic obesity is defined by concurrent low muscle strength and low muscle mass with high body fat[4]. Both sarcopenia and sarcopenic obesity are important but under-recognized factors influencing surgical outcomes and patient recovery after total joint arthroplasty (TJA). Ardeljan et al. [5] reported that individuals with sarcopenia had longer hospital stays, increased incidence of 90-day medical complications, and 2-year post-surgical implant-related complications after primary total knee arthroplasty compared to those without sarcopenia. Sarcopenia has been linked to increased risk of prosthetic loosening and urinary tract infection following both total knee and total hip arthroplasty, and additionally an increased risk of periprosthetic fracture, mechanical loosening, blood transfusion, and pneumonia following total knee arthroplasty[6]. Sarcopenia has also been found to be an independent predictor for prosthetic infection after TJA[7].

Unfortunately, sarcopenia and sarcopenic obesity do not appear to be rare in the hip and knee arthroplasty patient population, with prevalence reports ranging from 1 % to 72 % depending on the identification criteria used, age and comorbid conditions of the patients, including severity of osteoarthritis and other metabolic and inflammatory conditions[6,[8], [9], [10], [11], [12]]. A comprehensive understanding of the full impacts of these conditions on complication risk, recovery, and patient-satisfaction after TJA may still be unknown due to a lack of routine identification in research and clinical practice.

Sarcopenia and sarcopenic obesity may be reversible risk factors for surgical complications that could be optimized prior to elective TJA. Resistance exercise, nutritional support, treatment of related chronic diseases, and other interventions may improve muscle mass and strength before surgery and may mitigate potential risks of sarcopenia on total hip and knee arthroplasty outcomes[[13], [14], [15]]. Few studies have explored identification of these conditions in patients who present to an arthroplasty clinic. Consensus frameworks for identification of sarcopenia and sarcopenic obesity are available[16,17], but have not been widely evaluated in the context of real-world orthopedic clinical practice. In this pilot study we assessed the feasibility of screening and identifying these conditions in a hip and knee TJA clinic setting using published diagnostic frameworks.

## 2 Methods

**Fig. 1:**
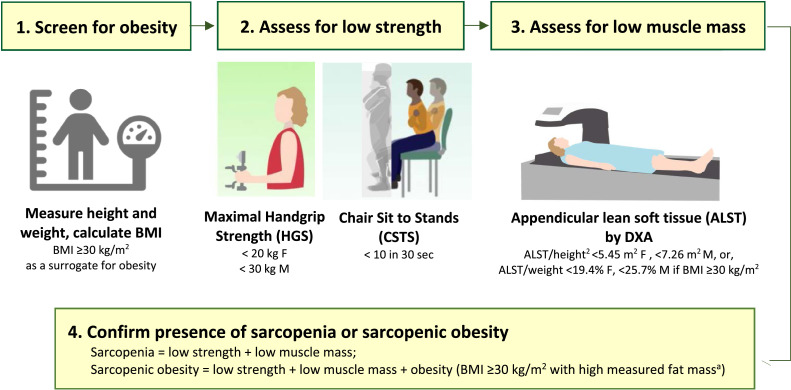
The framework used to assess for sarcopenia and sarcopenic obesity in the orthopedic clinic setting, based on the European Working Group on Sarcopenia in Older Persons (EWGSOP2)[16] and European Society for Clinical Nutrition and Metabolism and European Association for the Study of Obesity (ESPEN/EASO)[17]. ^a^DXA scan also enabled assessment of fat mass; all individuals with sarcopenic obesity had confirmed percent fat mass >40 % F, >30 % M[21]. *F* = female, *M* = male. Images adapted from the Noun Project, Adrien Coquet, and Prado CM, et al. Advances in Muscle Health and Nutrition: A Toolkit for Healthcare Professionals. Clinical Nutrition. 2022;41[10]:2244–2263.

This study was approved by our institutional review board. Individuals aged >40 years attending a weekly lower extremity arthroplasty clinic at a single tertiary care medical center in the Veterans Health Administration system from December 1, 2022 to February 28, 2023 were assessed for sarcopenia using the diagnostic framework from the European Working Group on Sarcopenia in Older Persons (EWGSOP2) criteria[16], or for sarcopenic obesity using the European Society for Clinical Nutrition and Metabolism and European Association for the Study of Obesity (ESPEN/EASO) framework[17] when body mass index (BMI) was ≥30 kg/m^2^, Fig. 1. The presence of both low strength and low muscle mass were required for identification of sarcopenia, and the presence of low strength, low muscle mass and high fat mass for sarcopenic obesity. Specific criteria are described below.

One clinic team member was dedicated to assessing patients for these conditions during routine clinic flow. The assessment process began with measurement of height and weight, and calculation of BMI. Individuals with a BMI <30 kg/m^2^ were assessed for sarcopenia, while those with a BMI >30 kg/m^2^ were considered for sarcopenic obesity. Screening proceeded with an assessment for low strength using maximal handgrip (HGS) and number of chair sit-to-stands in 30 s (CSTS), with each test taking <2 min of operational clinic time. HGS was assessed in both hands using a hydraulic handgrip dynamometer (Saehan Medical, Changwon, South Korea). Participants were seated with their elbow positioned at 90 degrees and instructed to squeeze the dynamometer with maximal effort. Three attempts were conducted in each hand, alternating between right and left to incorporate a rest period, and results were recorded to nearest 0.5 kg. The highest score from either hand was selected as maximal HGS. Participants then completed a 30-second CSTS. Beginning from a seated position in a standardized height chair with arms across their chest, participants stood up fully and then returned to a seated position. The maximum repetitions completed during a 30-second timed interval were recorded as CSTS. Low strength was confirmed by either low sex-specific HGS (<20 kg in females, <30 kg in males)[18] or a conservative cut-point of <10 CSTS in 30-seconds[19].

Patients who were identified as having low strength (either or both low HGS and low CSTS) were recommended to complete a body composition assessment through a same day appointment for dual-energy x-ray absorptiometry (DXA). DXA appointments were arranged through the radiology department adjacent to the orthopedic clinic in the same building. Low muscle mass was identified by appendicular lean soft tissue (ALST), the lean soft tissue of the arms plus legs. ALST was adjusted by height^2^ (for sarcopenia) or weight, % (for SO), and used as a surrogate for low skeletal muscle mass. Sex-specific cut-points were applied (ALST/height^2^ < 5.45 kg/m^2^ in females, <7.26 kg/m^2^ in males; ALST/weight <19.4 % females, <25.7 % males)[20]. Individuals who had both low strength + low muscle mass and a BMI <30 kg/m^2^ were confirmed to have sarcopenia. Individuals who had low strength + low muscle mass + obesity (BMI ≥30 kg/m^2^ and measured percent fat mass >40 % F, >30 % M[21]) were confirmed to have sarcopenic obesity. The workflow for this assessment is summarized in Fig. 1.

### 2.1 Statistical analysis

Descriptive analyses are reported as mean (standard deviation) and frequency (proportion), and feasibility was determined by the proportion of eligible participants able to complete assessment tests. Reasons for failure to complete the assessments were categorized[22] and reported as percentages.

## 3 Results

**Fig. 2:**
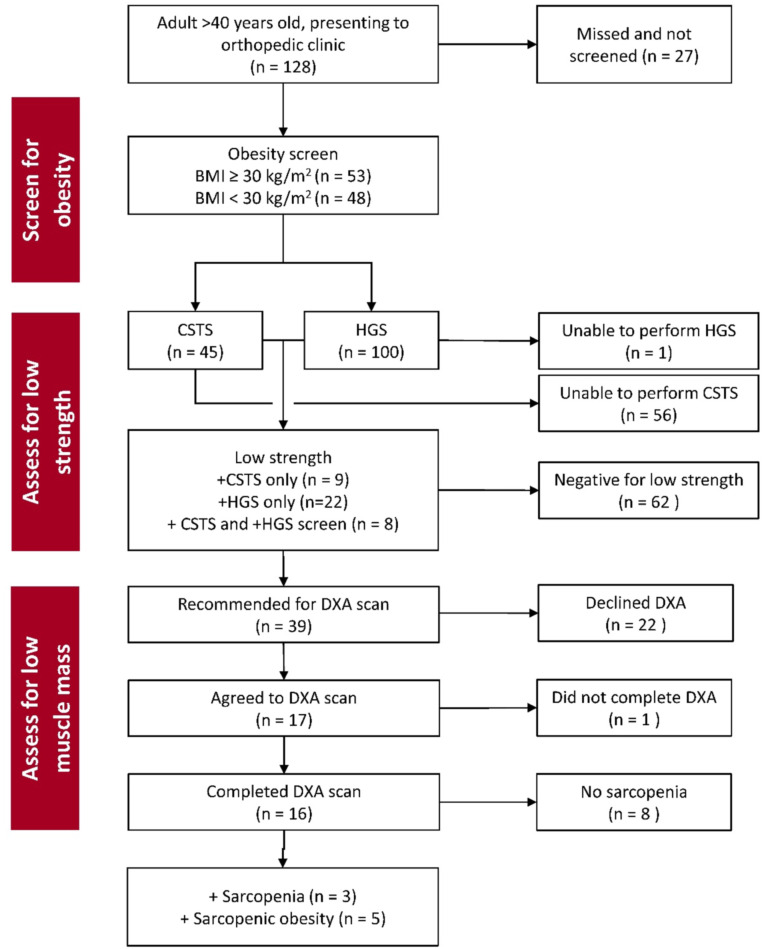
Flowchart of results of screening and identifying sarcopenia and sarcopenic obesity in patients attending the lower extremity arthroplasty clinic. BMI=body mass index, CSTS=chair sit-to-stands in 30 s, DXA=dual-energy x-ray absorptiometry, HGS=maximal handgrip strength.

There were 128 eligible patients who attended the orthopedic clinic during the five consecutive weekly clinics held between December 2022 - February 2023. Screening was completed in *N* = 101 patients (79 %) (Fig. 2)**.** The sample screened was 93 % male, mean age 69.6 ± 8.9 [range 47–96] years and BMI 31.7 ± 7.3 kg/m^2^. Half the sample (52 %) had a BMI ≥30 kg/m^2^. Reasons for clinic visit included new consults for knee or hip arthroplasty (*n* = 48), follow up from recent arthroplasty, <6 months (*n* = 21), follow up from prior arthroplasty, >1 year (*n* = 25), and other visit reasons (*n* = 7), including hip fracture follow-up and back pain.

Assessment for low HGS was completed in 99 % of patients (one had cervical radiculopathy impacting grip). The CSTS test was completed by only 44.5 % of patients (36 had non-completion due to joint pain, 7 for poor balance, 6 for inability to stand without pushing up using their hands, 3 for vertigo or hypotension, 1 for joint stiffness, and 3 for refusal, reasons not reported). Based on these tests, 39 patients (38.6 %) were determined to have low strength (low HGS=30, low CSTS=17, both low HGS+CSTS=8). These 39 patients were recommended for a same-day DXA scan. Seventeen agreed to stay and have the scan, but only sixteen completed as the DXA was unavailable for one patient. Twenty-two patients declined the DXA scan (15 reported being unable to stay, and 7 were uninterested). There were no differences in age or BMI in those completing or not completing the DXA scan. Among the 16 patients who completed all assessments, 3 had sarcopenia and 5 had SO, Fig. 2.

## 4 Discussion

Assessing for sarcopenia and sarcopenic obesity using current consensus frameworks may be challenging to achieve across all patients in a lower extremity arthroplasty clinic. In this pilot study, we were able to complete assessment of hand grip strength in only 79 % of eligible patients (101/128), even with a dedicated person conducting assessments during normal clinic flow. This suggests that changes in clinic flow or prioritization of these measures for prognostic identification of these relevant conditions may be important for this at-risk population of lower extremity arthroplasty patients.

Among the 101 patients assessed in this study, 39 had low strength (38.6 %), and 8 had either sarcopenia or sarcopenic obesity (7.9 %). Less than half of the patients with low strength who were recommended for DXA were able to stay and complete the scan. However, 50 % of those with all testing completed were found to either have sarcopenia or sarcopenic obesity. This suggests a potential high prevalence of these conditions that were not accurately captured in our pragmatic approach. The overall prevalence of sarcopenia or sarcopenic obesity identified by the EWGSOP2 or ESPEN/EASO criteria in this study was thus 6.25 % (8/128). This is likely a lower bound for the true prevalence of patients who had either of these conditions in our clinic given the incomplete screening in our study.

Even a 6.25 % prevalence of sarcopenia or sarcopenic obesity in a total joint arthroplasty population is not negligible. This suggests an opportunity for improving the risk profile for total joint arthroplasty patients prior to surgery within a reasonable time frame. Preoperative resistance exercise, in conjunction with dietary intervention and possibly other interventions, may be able to resolve low strength and muscle mass before elective surgery and potentially improve patient functional outcomes and lower the surgical risk profile for patients with sarcopenia or sarcopenic obesity, but this has yet to be determined.

Assessment for low strength relative to sarcopenia and sarcopenic obesity was feasible for nearly everyone we tested using HGS criteria, however CSTS was a less suitable metric for this patient population. This was due to high CSTS incompletion rates associated with lower extremity joint pain and stiffness in our study population. Whether it is necessary and relevant to assess both upper and lower extremity strength is not clear in current sarcopenia literature and may evolve with further global consensus efforts[3,23]. Currently, low HGS alone is sufficient to identify generalized muscle weakness relevant for sarcopenia and sarcopenic obesity identification. Physical performance measures like CSTS may be more appropriate as outcomes[23]. Notably, research on this topic is progressing and global leadership groups [i.e. Global Leadership Initiative in Sarcopenia (GLIS)[3], and the Sarcopenic Obesity Global Leadership Initiative (SOGLI)[24]] aim to improve operational definitions and standardized diagnostic criteria for clinical settings.

DXA completion was limited in our study to only 17 of 39 patients (44 %) who had low strength. Our lack of success in getting all the patients with low strength to complete the DXA scan was likely due to our focus on same-day DXA appointments. Offering the possibility of a DXA scan at a later date when it could be more convenient for the patient would be reasonable and likely improve uptake. There is typically ample time for patients who are being considered for lower extremity arthroplasty to have a DXA scan before their planned surgery date. This barrier could be overcome, and the percentage of patients having complete assessments for low muscle mass could be substantially improved.

This study has limitations. This was a preliminary approach to assess for sarcopenia and sarcopenic obesity in a clinical orthopedic setting using consensus frameworks. Though it is unlikely that the low strength testing would have had different results had we structured the study differently, expanded access for DXA scans to identify low muscle mass could have been more complete had we allowed for patients to return at their convenience for this assessment. This study was conducted in a single Veterans Affairs medical center, with a patient population that was predominantly male as expected in this setting. Patient sex, social circumstances, and clinic flow are likely to be different in other clinic settings and could lead to different results, impacting the generalizability of findings. Additionally, reasons for clinic appointment differed among patients, with some individuals post-TJA and others consulting for TJA. Although we were able to pragmatically examine identification frameworks for sarcopenia and sarcopenic obesity in a lower extremity arthroplasty clinic, our results need to be confirmed in a large study. Further studies on the impacts of sarcopenia and sarcopenic obesity on TJA-related health care system costs may be necessary to inform and guide clinical adoption of these frameworks in practice.

## 5 Conclusions

We identified that sarcopenia and sarcopenic obesity assessment in a lower extremity arthroplasty clinic using current frameworks is feasible but challenging to complete in all patients without adjustment to clinic flow and access to personnel and body composition scanning appointments. Despite failing to achieve comprehensive assessment of all patients in this feasibility study, we identified 6.25 % of patients with either sarcopenia or sarcopenic obesity. This is likely a lower bound for the true prevalence of these relevant clinical conditions and suggests an opportunity to intervene before surgery to improve total joint arthroplasty outcomes.

## Authors’ contributions

KG and NG conceptualized the study. IH, GL, and NG completed data collection. KG and NG analysed the data and drafted the initial manuscript. All authors revised the manuscript for intellectual content and have read and approved the final version.

## Funding

KG was funded by an Alberta Innovates Fellowship in Health Innovation, and a Mitacs Globalink Award for a visiting fellowship at Stanford University.

### Ethics approval

Provided by the Research Ethics Board at Stanford University.

## Declaration of competing interest

The authors declare the following financial interests/personal relationships which may be considered as potential competing interests:

Kristine Godziuk reports financial support was provided by Mitacs Inc. Kristine Godziuk reports financial support was provided by Alberta Innovates. If there are other authors, they declare that they have no known competing financial interests or personal relationships that could have appeared to influence the work reported in this paper.
